# Newly Proposed Diagnostic Criteria for Acute Respiratory Distress Syndrome: Does Inclusion of High Flow Nasal Cannula Solve the Problem?

**DOI:** 10.3390/jcm12031043

**Published:** 2023-01-29

**Authors:** Rong Liufu, Chun-Yao Wang, Li Weng, Bin Du

**Affiliations:** State Key Laboratory of Complex Severe and Rare Diseases, Medical ICU, Peking Union Medical College Hospital, Peking Union Medical College & Chinese Academy of Medical Sciences, Beijing 100730, China

**Keywords:** acute respiratory distress syndrome, high-flow nasal cannulas, diagnosis criteria

## Abstract

Acute respiratory distress syndrome (ARDS) is a common life-threatening clinical syndrome which accounts for 10% of intensive care unit admissions. Since the Berlin definition was developed, the clinical diagnosis and therapy have changed dramatically by adding a minimum positive end-expiratory pressure (PEEP) to the assessment of hypoxemia compared to the American-European Consensus Conference (AECC) definition in 1994. High-flow nasal cannulas (HFNC) have become widely used as an effective respiratory support for hypoxemia to the extent that their use was proposed in the expansion of the ARDS criteria. However, there would be problems if the diagnosis of a specific disease or clinical syndrome occurred, based on therapeutic strategies.

## 1. Introduction

Acute respiratory distress syndrome (ARDS) is defined as an acute diffuse, inflammatory lung injury, leading to increased pulmonary vascular permeability, increased lung weight, and loss of aerated lung tissue [1]. Since the first description of ARDS by Ashbaugh, in 1967 [2], the consensus on diagnostic criteria has not been reached until the development of the American-European Consensus Conference (AECC) definition in 1994 [3]. In 2012, the Berlin definition of ARDS was developed [1], to address the limitations of the AECC definition, including the inciting cause, time frame of acute onset, minimum requirement of positive end-expiratory pressure (PEEP), interpretation of chest radiograph, and the origin of pulmonary edema. Although the limitations of the prior consensus have been recognized, the current definition is still subjected to the lack of specific diagnostic tests, and difficulty in standardizing the PaO_2_/FiO_2_ ratio since it varies with FiO_2_, PEEP, and timing. The fundamental challenge lies in the diagnostic criteria that may preclude the accurate and standardized definition of ARDS, which affects the incidence and later therapeutic strategy.

The incidence of ARDS varies widely by countries and regions, ranging from 10.1 to 78.9 per 100,000 person-years [4,5,6]. Recent data about the epidemiology of ARDS, based on the Berlin definition, came from the large observational study to understand the global impact of severe acute respiratory failure (LUNG SAFE) study, an international, multicenter, prospective cohort study of 29,144 patients undergoing invasive or noninvasive ventilation during 4 consecutive weeks in the winter of 2014 in a convenience sample of 459 intensive care units (ICUs) from 50 countries across 5 continents [7]. Although the true geographic variation might affect the variability in the incidence of ARDS, it also revealed the methodologic differences in how the definition of ARDS was applied and the availability of ICU resources. ARDS represented 0.42 cases per ICU bed over 4 weeks, equivalent to 10.4% (95% confidence interval [CI] 10.0–10.7%) of ICU admissions and 23.4% of patients requiring mechanical ventilation, 40.0% of whom died before hospital discharge [7]. Despite the disease burden (including morbidity and mortality) of ARDS among critically ill patients, the fundamental challenge exists in the lack of a specific diagnostic test, leading to the ongoing controversy in the definition and diagnostic criteria for ARDS.

## 2. PEEP Dilemma in Oxygenation Criteria

In the AECC definition, hypoxemia was defined as PaO_2_/FiO_2_ ratio ≤300 mmHg, regardless of PEEP [3]. The panel argued that, although PEEP could exert a profound impact on pulmonary shunt fraction, the response to PEEP was time dependent and highly individualized. As a result, PEEP was left out of the oxygenation criteria [3] (Table 1).

AECC, American-European Consensus Conference; ARDS, acute respiratory distress syndrome; FiO_2_, fraction of inspired oxygen; HFNC, High-flow nasal cannula; PaO_2_, arterial partial pressure of oxygen; PEEP, positive end-expiratory pressure; SpO_2_, peripheral capillary oxygen saturation.

In the Berlin definition, while acknowledging that PEEP could markedly affect PaO_2_/FiO_2_ ratio, a minimum PEEP or continuous positive airway pressure (CPAP) level of 5 cm H_2_O, which could be delivered by non-invasive ventilator (NIV) in mild ARDS, was included in the diagnostic criteria [1]. However, the CPAP requirement, which could be delivered by non-invasive ventilation (NIV), was only allowed in the diagnosis of mild ARDS, indicating that patients undergoing NIV could not be categorized as moderate or severe ARDS. In a secondary analysis of LUNG SAFE study, Bellani et al. reported that rates of NIV use were similar between the mild, moderate, and severe ARDS groups (14.3, 17.3 and 13.2%, respectively), while mortality rates were not (22.2, 42.3, and 47.1%, respectively) [8]. These results suggested that ARDS of any severity could be classified in patients receiving NIV by the use of PaO_2_/FiO_2_ bands.

However, PEEP is used in an unpredictable fashion, not only in clinical practice, but also during the process of development of consensus definition. A minimum PEEP level of 10 cm H_2_O was also proposed and empirically evaluated for the severe ARDS category [1]. Under standardized ventilator settings (i.e., PEEP ≥ 10 cm H_2_O and FiO_2_ ≥ 0.5), Villar et al. identified a subset of more severe patients, with very high hospital mortality (67.0%), based on PaO_2_/FiO_2_ ratio assessed 24 h after ARDS onset [9], suggesting the need for a new standardized method for evaluating oxygenation criteria [10]. Of note, in the patient-level meta-analysis of 4457 patients with ARDS evaluating the Berlin definition, PEEP ≤ 10 cm H_2_O and other ancillary variables (severity of chest radiograph, static compliance of the respiratory system [CRS] ≤ 40 mL/cm H_2_O, corrected expired minute ventilation [VECORR] ≥ 10 L/min), in addition to oxygenation, did not identify a group of patients with higher mortality, and were excluded from the final Berlin definition [1]. To make things more complicated, even the same PEEP level per se might produce quite different transpulmonary pressure levels in different patients, partly attributable to the underlying (pulmonary vs. extrapulmonary) diseases [11], indicating that setting the same PEEP level would still result in a non-standardized condition.

## 3. Inclusion of HFNC as a Modification of Berlin Definition

Since 2015, the high-flow nasal cannula (HFNC) has become widely used as an effective respiratory support for acute hypoxemic respiratory failure (AHRF). The high flow minimizes entrainment of room air, thereby maintaining a precision FiO_2_. It also flushes out expired gas from the nasopharyngeal dead space. The built-in heat humidifier improves patient comfort and tolerance with warmed and humidified gas. In addition, HFNC also impedes expiratory flow, producing distending pressure similar to CPAP or PEEP [12], with an increase in hypopharyngeal pressure by about 1 cm H_2_O per 10 L/min flow [13].

Based on the positive results from many clinical trials, the European Respiratory Society (ERS) issued a clinical practice guideline, which suggested the use of HFNC in patients with AHRF, during breaks from NIV in patients with AHRF, and in postoperative or nonsurgical patients after extubation [14].

During the coronavirus disease 2019 (COVID-19) pandemic, the use of HFNC in patients with severe COVID-19 was shown to be associated with a reduced need for endotracheal intubation, despite no impact on hospital mortality [15,16]. For example, in a randomized, open-label clinical trial of 220 adult patients with severe COVID-19, defined as PaO_2_/FiO_2_ ratio <200 mmHg, 34 (34.3%) of 99 patients randomized to HFNC and 51 (51.0%) of 100 patients randomized to conventional oxygen therapy required endotracheal intubation within 28 days (hazard ratio [HR], 0.62; 95% CI, 0.39–0.96; *p* = 0.03). The median time to clinical recovery, another component of co-primary outcomes, was 11 (interquartile range [IQR], 9–14) days in the HFNC group vs. 14 (IQR, 11–19) days in the conventional oxygen therapy group (HR 1.39; 95% CI, 1.00–1.92; *p* = 0.47). However, the mortality rate at day 28 was 8.1% (8/99) in the HFNC group, compared with 16.0% (16/100) in the conventional oxygen therapy group (HR, 0.49; 95% CI, 0.21–1.16; *p* = 0.11) [15]. In another prospective randomized clinical trial of 711 patients with respiratory failure due to COVID-19 in 34 ICUs in France, the 28-day all-cause mortality rate, the primary endpoint, was 10% (36/357) with HFNC and 11% (40/354) with standard oxygen therapy (absolute difference, −1.2% [95% CI, −5.8% to 3.4%]; *p* = 0.60), while the endotracheal intubation rate was significant lower with HFNC than with standard oxygen therapy (45% [160/357] vs. 53% [186/354]; absolute difference, −7.7% [95% CI, −14.9% to −0.4%]; *p* = 0.04) [16]. As a result, HFNC was recommended by the international and national guidelines issued by the Surviving Sepsis Campaign, the ERS, and National Institute of Health (NIH) [14,15,16,17,18,19], although conflicting results also existed [20].

Given the increasing use of HFNC in the management of AHRF due to a variety of etiologies, some investigators proposed that the Berlin definition of ARDS be expanded to include patients treated with HFNC with at least 30 L/min who fulfilled the other criteria for the Berlin definition of ARDS [21,22,23,24]. Such an expanded definition was believed to facilitate the diagnosis of ARDS in a timely fashion and in a wider patient population, expanding to patients with mild-to-moderate lung injury who required a certain level of respiratory support, regardless of the need for endotracheal intubation and/or positive-pressure ventilation [22]. It was also believed that this expanded definition of ARDS would also help patient management in clinical practice and patient recruitment in clinical research.

## 4. Inclusion of HFNC Does Not Solve the Problems with the Berlin Definition

Both the Berlin definition and the proposed modification are subject to an overt limitation, in which the severity of hypoxemia was assessed by a certain mode of respiratory support (such as HFNC, NIV, and invasive mechanical ventilation (IMV)) [1,22]. As a matter of fact, the majority of the clinical diseases (e.g., severe acute pancreatitis) or clinical syndromes (e.g., circulatory shock) are not, and should not be, diagnosed or defined according to the therapeutic intervention.

However, to the best of our knowledge, this was not without precedent. For example, in the definition of multiple system organ failure proposed by Fry et al., pulmonary failure was defined as hypoxia that warranted respirator-assisted ventilation for at least 5 days postoperatively or until death [25]. In the sequential (sepsis-related) organ failure assessment (SOFA) score [26], which is also used in the sepsis-3 consensus definition [27], the severity of the respiratory system dysfunction is defined based on the use of respiratory support, while the use of catecholamines is included in the evaluation of cardiovascular dysfunction. A similar example is the diagnostic criteria of polymyalgia rheumatica (PMR), which required a “rapid response” to low-dose corticosteroid therapy in the early definition [28]. One of the major debates is whether a response to corticosteroids should be included in the diagnostic criteria due to the lack of consensus with regard to the standardized dose, the route, and the duration of corticosteroid therapy, as well as the standard definition of “rapid response” (Table 2). Thus, the response to corticosteroid therapy was removed from the 2012 provisional classification criteria for PMR by the European League Against Rheumatism and the American College of Rheumatology (EULAR/ACR) [29].

Acknowledging that PMR is a disease whereas ARDS is a clinical syndrome, both diagnostic criteria do share the following common issue. If we want to define a disease or clinical syndrome according to any therapeutic interventions and/or a minimal response to a therapeutic intervention, it should be based on two premises: first, all patients with the disease/syndrome should have the same chance of receiving the specified therapeutic intervention; second, all clinicians may comply to the same strategy with regard to the specified therapeutic intervention (e.g., PEEP setting). When applying the above two premises to patients with ARDS, this means that all patients with ARDS should have the same chance of receiving the same respiratory support (including HFNC, NIV, and IMV), and all intensive care physicians should set the same PEEP level in the same patient.

Unfortunately and obviously, none of the above premises is true. The LUNG SAFE study observed a pooled incidence of ARDS of 0.42 cases/ICU bed over 4 weeks, but with significant geographic variation in Oceania (0.57), Europe (0.48), North America (0.46), Africa (0.32), South America (0.31), and Asia (0.27) [7]. However, these findings might be interpreted in the light of the different geographic distribution of critical care resources [30]. Arabi et al. observed considerable variation in critical care resources in 20 countries across Asia [31]. For example, there were 0.18 noninvasive ventilators and 0.72 invasive ventilators per ICU bed in high-income countries (HICs), compared with 0.12 and 0.42 in low-income countries (LICs). This suggested that patients with AHRF in LICs might not have the same chance of receiving invasive or noninvasive mechanical ventilation as those in HICs, while, according to the Berlin definition, those who were not treated with mechanical ventilation did not meet the diagnostic criteria for ARDS. In other words, the countries with a high number of ICU beds and ventilation assistance would label ARDS cases that are probably not able to receive this level of resources in other countries. Furthermore, there are multiple methods of optimal PEEP selection in ARDS (PEEP-FiO_2_ table, recruitment maneuver, pressure-volume curve analysis, maximal static compliance, optimal driving pressure, lowest intrapulmonary shunt, minimal PaCO2-to-end-tidal CO2 gradient, transpulmonary pressure, lung computed tomography and electronic impedance tomography), all of which have pros and cons, while the best approach remains unknown [32]. As a result, PEEP selection is highly variable, and clinician specific in clinical practice, even in the same patient.

The call for modification of the Berlin definition reflects the pitfalls of current practice, i.e., defining a disease/syndrome according to a specified therapeutic intervention. However, the inclusion of HFNC will not solve the above problems, because the game rule remains unchanged. Let us assume a patient with AHRF who is treated with awake extracorporeal membrane oxygenation (ECMO) but breathing room air. This patient does not meet the current Berlin definition or proposed modified definition of ARDS. In this way, shall we propose another modification of the Berlin definition to include patients treated with ECMO?

In addition, some investigators argued that the diagnostic criteria should include some direct measure of lung injury specific to ARDS, such as increased extravascular lung water, dead space fraction, or a direct measure of permeability, while acknowledging the feasibility issues [33]. During recent years, combined clinical and biological data have been used to identify two phenotypes across different ARDS cohorts, termed hyper- and hypo-inflammatory [34]. These biologically derived phenotypes have widely divergent clinical outcomes and a differential response to higher PEEP level [35], restrictive fluid management strategy [36], and statin treatment [37] in the secondary analysis of completed trials. This novel classification of ARDS based on biological phenotyping, with the use of latent class analysis, may shed light on the understanding of the inflammatory pathophysiology of ARDS, leading to further modification of the diagnostic criteria of ARDS in the future.

## 5. Conclusions

We believe that the modified ARDS definition should exclude the PEEP requirement from the oxygenation criteria, as the Kigali modification [38] (Table 1). Thus, the diagnosis of hypoxemia will be independent of the need for any type of respiratory support. Moreover, we also strongly believe that the diagnostic criteria for any disease/syndrome should be based on pathophysiology, not prognostic value.

## Figures and Tables

**Table 1:** The different requirements of PEEP in definition and modifications of ARDS criteria.

	AECC Definition	Berlin Definition	Kigali Modification	Matthay Modification
Timing	Acute	1 week	1 week	1–2 weeks
Oxygenation	PaO_2_/FiO_2_ ≤ 300 mmHg	PaO_2_/FiO_2_ ≤ 300 mmHg	SpO_2_/FiO_2_ ≤ 315	PaO_2_/FiO_2_ ≤ 300 mmHg or SpO_2_/FiO_2_ ≤ 315
Chest radiograph	Bilateral opacities	Bilateral opacities, with radiograph criteria and examples	The same as Berlin definition	Opacities in two quadrants (bilateral or unilateral) or ultrasonography scan
Origin of pulmonary edema	PAWP ≤ 18 mmHg when measured or no clinical evidence of left atrial hypertension.	Respiratory failure not fully explained by cardiac failure or fluid overload	The same as Berlin definition	The same as Berlin definition
Risk factors	None	Specific criteria	The same as Berlin definition	The same as Berlin definition
PEEP requirement	No requirement	PEEP ≥ 5 cm H_2_O with invasive ventilation (non-invasive ventilation in the mild category.)	No requirement	PEEP/CPAP ≥ 5 cm H_2_O or HFNC ≥ 30 L/min
Reasons for PEEP requirement	PEEP is time dependent and highly individualized	PEEP can markedly affect PaO_2_/FiO_2_	The same as AECC	HFNC ≥ 30 L/min provided similar PEEP (2–5 cm H_2_O)
Limitations	Failure to define sensitivity of PaO_2_/FiO_2_ to different ventilator settings	Misdiagnosis of patients without chance for assistant ventilator	The same as AECC	Misdiagnosis from non-standardization of different intensivists.

**Table 2:** Therapeutic interventions in diagnostic criteria: response to corticosteroid therapy in polymyalgia rheumatica versus PEEP requirement in ARDS.

	Polymyalgia Rheumatica	ARDS
Therapeutic intervention	Response to corticosteroids therapy	PEEP requirement
First appearance in diagnostic criteria (year)	Healey criteria (1986)	Berlin definition (2012)
Dose	Prednisone 20 mg equivalent dose or less per day	5 cm H_2_O or more
Route of administration	Not defined	CPAP, HFNC or MV
Therapeutic response	A patient-reported global improvement of 70% within a week of commencing corticosteroids and normalization of inflammatory markers within 4 weeks. A lesser response should encourage the search for an alternative condition	PaO_2_/FiO_2_ ≤ 300 mmHg
Unanswered questions	Level of response undefined; Time frame of response poorly defined; Response to corticosteroids highly individualized	Response to PEEP is time dependent; Response to PEEP is highly individualized
Limitations	No uniform response to corticosteroids in patients with PMR; Patients with inflammatory diseases other than PMR may also respond to corticosteroids	No uniform access to respiratory support in different geographic regions; No consensus on PEEP selection

## Data Availability

Not applicable.
